# Assessment of Urinary Betaine as a Marker of Diabetes Mellitus in Cardiovascular Patients

**DOI:** 10.1371/journal.pone.0069454

**Published:** 2013-08-06

**Authors:** Hall Schartum-Hansen, Per M. Ueland, Eva R. Pedersen, Klaus Meyer, Marta Ebbing, Øyvind Bleie, Gard F. T. Svingen, Reinhard Seifert, Bjørn E. Vikse, Ottar Nygård

**Affiliations:** 1 Section for Cardiology, Department of Clinical Science, University of Bergen, Bergen, Norway; 2 Nordic Centre of Excellence in Human Nutrition – MitoHealth, University of Bergen, Bergen, Norway; 3 Department of Heart Disease, Haukeland University Hospital, Bergen, Norway; 4 Section for Pharmacology, Department of Clinical Science, University of Bergen, Bergen, Norway; 5 Laboratory of Clinical Biochemistry, Haukeland University Hospital, Bergen, Norway; 6 Bevital AS, Bergen, Norway; 7 Renal Research Group, Department of Clinical Science, University of Bergen, Bergen, Norway; 8 Department of Medicine, Haukeland University Hospital, Bergen, Norway; Baker IDI Heart and Diabetes Institute, Australia

## Abstract

Abnormal urinary excretion of betaine has been demonstrated in patients with diabetes or metabolic syndrome. We aimed to identify the main predictors of excretion in cardiovascular patients and to make initial assessment of its feasibility as a risk marker of future diabetes development. We used data from 2396 patients participating in the Western Norway B-vitamin Intervention Trial, who delivered urine and blood samples at baseline, and in the majority at two visits during follow-up of median 39 months. Betaine in urine and plasma were measured by liquid-chromatography-tandem mass spectrometry. The strongest determinants of urinary betaine excretion by multiple regression were diabetes mellitus, age and estimated glomerular filtration rate; all p<0.001. Patients with diabetes mellitus (n = 264) had a median excretion more than three times higher than those without. We found a distinct non-linear association between urinary betaine excretion and glycated hemoglobin, with a break-point at 6.5%, and glycated hemoglobin was the strongest determinant of betaine excretion in patients with diabetes mellitus. The discriminatory power for diabetes mellitus corresponded to an area under the curve by receiver-operating characteristics of 0.82, and betaine excretion had a coefficient of reliability of 0.73. We also found a significant, independent log-linear relation between baseline betaine excretion and the risk of developing new diabetes during follow-up. The good discriminatory power for diabetes, high test-retest stability and independent association with future risk of new diabetes should motivate further investigation on the role of betaine excretion in risk assessment and long-term follow-up of diabetes mellitus.

## Introduction

Betaine in plasma and its excretion in urine have recently gained attention as potential risk factors and markers of disease in humans. Plasma betaine is low in patients with dyslipidemia [1] and is also inversely associated with other components of the metabolic syndrome [2]. Lever et al [3] demonstrated years ago that many patients with diabetes had a substantial increase in urinary betaine excretion, which was associated with hyperglycemia and proximal tubular dysfunction [4]. Both plasma betaine and urinary excretion show high test-retest reliability in (smaller) longitudinal studies [5], [6], [7].

Betaine is a quaternary ammonium compound, which is obtained from foods or is synthesized through mitochondrial oxidation of choline [8]. In the kidney it is freely filtered in the glomeruli and actively reabsorbed in the renal tubuli. Fractional reabsorption is normally very high (>98%) [9] and may involve several tubular transport mechanisms [10], [11].

In mammalian physiology betaine has two main functions. It is a major osmolyte involved in cell volume regulation, accumulating to high concentration in many tissues [12]. This function may be particularly important in the kidneys, where betaine preserves tissue integrity and protects the medullary cells against hypertonicity. Betaine also serves as a methyl donor in a reaction converting homocysteine to methionine, catalysed by the enzyme betaine-homocysteine methyltransferase (BHMT) [13], which is expressed at a very high level in human liver and kidney. BHMT activity is increased in type 2 diabetic rats [14] and has ramifications for lipid metabolism, thereby linking lipid and one-carbon metabolism [2], [15].

The role of betaine in renal function and the high excretion in diabetic patients motivate large clinical studies on determinants of urine betaine to evaluate its potential as a diagnostic marker. The present study is based on data from the Western Norway B-vitamin Intervention Trial (WENBIT), investigating the secondary prevention of cardiovascular events with B-vitamins in patients with mainly established ischemic heart disease.

## Methods

### Ethics Statement

The study was approved by the Regional Committee for Medical and Health Research Ethics, the Data Inspectorate, and the Norwegian Directorate of Health.

### Study population

WENBIT included a total of 3090 patients undergoing coronary angiography for suspected coronary artery disease in 1999–2004, of whom 89% had stable angina pectoris [16]. The primary objective of the study was to investigate whether homocysteine-lowering treatment with folic acid and vitamin B12 could reduce cardiovascular events and mortality in these patients. Details on randomization and the vitamin regimen have been published elsewhere [16]. Because urinary creatinine levels are increased by tubular secretion in renal failure [17], [18], patients (n = 54) with estimated glomerular filtration rate (eGFR) <30 mL/min/1.73 m^2^ or macroalbuminuria (urinary albumin/creatinine ratio (UACR) >30 mg/mmol) were excluded from the present study.

Clinical information and blood and urine samples were collected at baseline, at a follow-up visit one year after randomization, and at a final study visit after median (IQR) 39 (17) months. A total of 2396 patients donated urine samples at baseline and met the inclusion criteria for the current investigation. Altogether, 1772 patients provided urine at all three visits, 348 patients at two visits, and 276 only at baseline. Blood samples were also drawn from the majority at a visit one month after randomization. Data on demographics, clinical conditions, lifestyle and medication were collected, as reported previously [16]. Diabetes mellitus was defined as previously diagnosed type 1 or type 2 diabetes. Diuretics included furosemide, thiazide, amiloride and spironolactone. Estimated glomerular filtration rate (eGFR) was calculated using the modified Chronic Kidney Disease Epidemiology Collaboration (CKD-EPI) equation [19]. Urinary albumin/creatinine ratio (UACR) was calculated as the ratio of spot urinary albumin to urinary creatinine, measured in mg/mmol. Fractional excretion was calculated as (urine betaine/plasma betaine)/(urine creatinine/serum creatinine)*100%.

Blood was usually drawn before 12:00 hours; at baseline, 764 (31.9%) participants were fasting. Blood and urine samples were immediately frozen at −80°C until analysis. Spot urine samples were first morning void in 1859 (77.6%) patients at baseline, 1456 (65.5)% at one year and 1550 (65.1%) at the end of the study.

Development of new diabetes during follow-up was assessed among patients (n = 2076) with no diagnosis of diabetes and either fasting glucose <7.0 mmol/L or non-fasting glucose <11.1 mmol/L at baseline. We defined new diabetes endpoints as (1) self-reported diabetes or (2) self-reported diabetes and/or fasting glucose ≥7.0 mmol/L or non-fasting glucose ≥11.1 mmol/L.

### Laboratory methods

Measurements of metabolites in plasma and urine were performed at the laboratory of Bevital AS (www.bevital.no). Betaine, choline, dimethylglycine and creatinine in EDTA-plasma were measured by LC-MS/MS [20] and total homocysteine by GC-MS/MS [21]. Betaine, choline, dimethylglycine and creatinine in urine were determined by a slight modification of the method based on LC-MS/MS [20], originally developed for plasma analysis. Sarcosine in urine was analyzed by GC-MS/MS. The amount of sarcosine in EDTA-plasma could not be determined due interference from sarcosine in the EDTA Vacutainer Tubes. The amount of urine metabolites are given as mmol per mol urinary creatinine. Glycated hemoglobin (HbA1c) was determined by matrix-assisted laser desorption/ionization time-of-flight mass spectrometry [22].

### Statistical analyses

We used baseline data for the assessment of determinants of urinary betaine excretion and available data from all three visits for longitudinal analyses. Data are presented as median (IQR). To test for sex differences we used Mann-Whitney-U test on scaled parameters and chi-square on nominal data. Associations were studied by Spearman correlations, linear regression and generalized additive models (GAM). In regression analyses, serum triglycerides, urinary albumin/creatinine ratio (UACR) and urinary betaine excretion were log-transformed because of skewness. The validity of the models was assessed by inspection of residual plots and Cook's distances. Unless otherwise specified, and in order to avoid over adjustment, HbA1c and serum glucose were omitted from models testing the effect of diabetes mellitus. In order to further study the influence of glucose metabolism, we divided the study population into three subgroups i.e. patients without diabetes with plasma glucose <5.5 mmol/L, patients without diabetes with plasma glucose ≥5.5 mmol/L, and patients with diabetes mellitus type 1 or type 2. Antidiabetic mono and combination therapy were coded as dummy variables. The distribution of betaine excretion was illustrated with Gaussian kernel density plots. We used GAM regression to obtain a graphical presentation of the non-linear relationships and segmented regression to identify possible break-points. All plots were made using R. Area under the curve (AUC) was derived from receiver operating curve (ROC) analysis. The test-retest reliability of metabolites over time was calculated in patients with at least two urine samples (n = 2120), as coefficient of reliability (CoR), estimated from linear mixed models. Odds ratios of new diabetes were estimated by multivariate logistic regression. Statistical analysis was done with SPSS version 18 (SPSS Inc., Chicago, Illinois) and R version 2.13.2 (R Foundation, Vienna, Austria). P-values <0.05 were considered statistically significant.

## Results

### Characteristics of the study population

The median (IQR) age was 62.0 (14.0) years and 490 (20.5%) were female (Table 1). Women were about 4 years older than men and more frequently received diuretics (23.7 vs. 13.2%). Men had more often suffered from acute myocardial infarction and had more extensive cardiovascular disease. The prevalence of diabetes was similar for the two genders (10.7% in men and 12.2% in women). Seventeen subjects had diabetes type 1. The median (IQR) urinary excretion of betaine was similar in men (7.3 (8.1) mmol/mol creatinine) and women (7.4 (7.7) mmol/mol creatinine). The distribution was skewed (kurtosis of 26), with levels exceeding 10 times the median value in 1.0% of women and 2.2% of men.

**Table 1:** Baseline characteristics.

	Men (n = 1906)	Women (n = 490)	P value
	Median (IQR)	Median (IQR)	
Age (years)	61.0 (13.0)	65.0 (15.0)	<0.001
Body mass index (kg/m^2^)	26.5 (4.3)	26.2 (5.9)	0.039
Smoking (%)	26.9	26.3	0.864
Medical history (%)			
Diabetes mellitus (type 1 and 2)	10.7	12.2	0.332
Diabetes mellitus type 2	9.9	11.8	0.244
AMI	42.8	33.1	<0.001
PCI	21.6	17.3	0.039
CABG	14.9	9.2	0.005
Hypertension	45.4	51.8	0.011
Diagnosis at inclusion (%)			0.158
Stable angina	90.6	92.7	
Acute coronary syndrome	9.4	7.3	
Left ventricular ejection fraction (%)	65 (11)	68 (10)	<0.001
Angiographic extent of CAD (%)			<0.001
0	9.5	18.4	
1	27.3	34.9	
2	27.0	25.1	
3	36.2	21.6	
Medication (%)			
Statin at discharge	88.7	86.1	0.136
Diuretics	13.2	23.7	<0.001
Metformin	3.8	5.1	0.198
Sulfonylurea	3.5	4.1	0.588
Insulin	3.0	3.5	0.662
Blood indices			
Total cholesterol (mg/dL)	186 (54)	197 (62)	<0.001
LDL-cholesterol (mg/dL)	112 (50)	116 (54)	0.016
HDL-cholesterol (mg/dL)	46.4 (15.5)	54.1 (19.3)	<0.001
Triglycerides (mg/dL)	142 (97)	124 (78)	<0.001
CRP (mg/L)	1.8 (2.9)	2.0 (3.5)	0.022
HbA1c (%)	5.8 (1.4)	5.8 (1.6)	0.36
eGFR (mL/min/1.73 m^2^)	93.2 (17.6)	89.0 (20.2)	<0.001
Chronic kidney disease stage 3 (eGFR 30–59)%	2.9	9.6	<0.001
Glucose (mmol/L)	5.7 (2.0)	5.5 (2.0)	0.002
Betaine ( µmol/L)	39.3 (15.7)	33.2 (15.6)	<0.001
Choline ( µmol/L)	9.6 (3.2)	9.1 (2.7)	<0.001
Dimethylglycine ( µmol/L)	4.1 (1.5)	3.7 (1.7)	<0.001
Total homocysteine ( µmol/L)	10.4 (3.6)	9.8 (3.7)	<0.001
Urinary compounds			
UACR (mg/mmol)	0.58 (0.70)	0.65 (0.89)	0.001
Betaine (mmol/mol creatinine)	7.3 (8.1)	7.4 (7.7)	0.562
Choline (mmol/mol creatinine)	1.8 (1.1)	2.3 (1.4)	<0.001
Dimethylglycine (mmol/mol creatinine)	3.2 (3.0)	3.4 (2.8)	0.084
Sarcosine (mmol/mol creatinine)	0.14 (0.13)	0.14 (0.12)	0.626

Abbreviations: IQR interquartile range; AMI acute myocardial infarction; PCI percutaneous coronary intervention; CABG coronary artery bypass graft; CAD coronary artery disease (number of diseased vessels); LDL low density lipoprotein; HDL high density lipoprotein; CRP C-reactive protein; HbA1c glycated hemoglobin; eGFR estimated glomerular filtration rate; UACR urinary albumin/creatinine ratio.

### Determinants of urinary betaine excretion

We initially investigated associations between betaine excretion and baseline characteristics by simple (adjusted for age and sex) and multiple linear regression analyses (Table 2). In multiple regression, diabetes mellitus, age, and eGFR were the strongest determinants of betaine excretion, in that order, all p<0.001 (Table 2). Notably, inclusion of product terms in the regression model demonstrated negative interaction between diabetes and plasma betaine and between diabetes and use of diuretics, and a positive interaction between diabetes and eGFR, all p<0.001.

**Table 2:** Determinants of urinary betaine excretion at baseline by linear regression.

	Adjusted for gender and age			Multivariate^a^			
	β^b^	P value		β^b^		P value	
Gender (male)	0.01	0.58		−0.01		0.57	
Age	0.13	<0.001		0.17		<0.001	
Body mass index	0.15	<0.001		0.07		0.001	
Smoking (yes)	−0.11	<0.001		−0.11		<0.001	
Diabetes mellitus (yes)	0.42	<0.001		0.38		<0.001	
Hypertension (yes)	0.09	<0.001		0.01		0.66	
eGFR	0.12	<0.001		0.14		<0.001	
Serum HDL cholesterol	−0.09	<0.001		−0.03		0.14	
Serum triglycerides^c^	0.10	<0.001		0.02		0.36	
Plasma betaine	0.01	0.60		0.07		<0.001	
UACR^c^	0.17	<0.001		0.10		<0.001	
Explained variance (R^2^)					0.23		

Abbreviations: eGFR estimated glomerular filtration rate; UACR urinary albumin/creatinine ratio.

a Adjusted for all variables in the model.

b Standardized beta-coefficient.

c Log transformed.

Not significant in age and gender adjusted analysis: Acute coronary syndrome, diuretics, ACE-inhibitors, fasting blood sample, N-vessel disease, total cholesterol, low density lipoprotein, apo lipoprotein A1, apo lipoprotein B, CRP.

### Urinary betaine excretion according to diabetes mellitus and serum glucose

We compared betaine excretion and its associations with selected clinical and metabolic indices in subjects with and without previously diagnosed diabetes. Patients without diabetes were divided into two groups, without regard to fasting status, with serum glucose <5.5 (n = 978) and ≥5.5 (n = 1154) mmol/L, referred to as low and high glucose groups, respectively.

**Figure 1:**
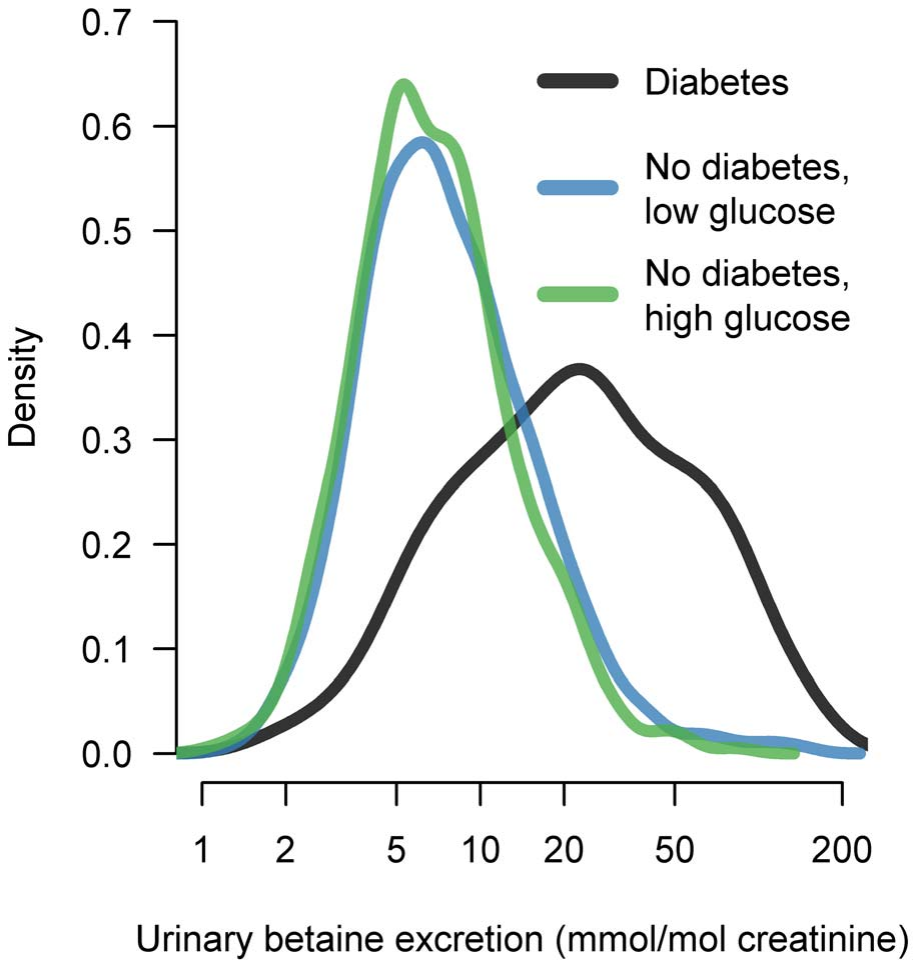
Density plot showing the relative distributions of urinary betaine excretion in patients with diabetes (black line), no diabetes and serum glucose ≥5.5 mmol/L (green line), and no diabetes and serum glucose <5.5 mmol/L (blue line).

The median (IQR) betaine excretion was more than three times higher in patients with diabetes (22.2 (35.7) mmol/mol creatinine) compared with patients without diabetes (p<0.001), who showed a small but significant difference in excretion according to low (6.5 (5.7) mmol/mol creatinine) or high serum glucose (7.1 (7.0) mmol/mol creatinine), (p = 0.003). The distributions are shown in figure 1. The median (IQR) fractional betaine clearance in these three groups were 4.6 (7.7)%, 1.2 (1.2)% and 1.4 (1.5)%. Excretion of betaine did not differ between patients with type 2 (21.2 (36.0) mmol/mol creatinine) and type 1 (26.7 (28.9) mmol/mol creatinine) diabetes (p = 0.28). Excretion of related methylamines, like choline, dimethylglycine and sarcosine across the three patient groups showed similar but weaker differences (ratios between median excretion in patients with diabetes and patients with low glucose were 1.5, 1.8 and 1.9, respectively), whereas excretion of the amino acids glycine and serine were essentially the same between the three subgroups. For the entire cohort, using univariate Spearman analyses, urinary betaine was strongly correlated with urinary dimethylglycine (rho = 0.75) and sarcosine (rho = 0.75), and moderately correlated with urinary choline, glycine and serine (rho = 0.46, 0.32 and 0.40, respectively), all p<0.001. The relation between urinary excretion of betaine and the other amines were similar in all three subgroups.

The associations of betaine excretion with several clinical and metabolic indices showed remarkable differences across the three groups, with the largest contrasts when comparing patients with diabetes and patients with no diabetes and low glucose (Table 3). In diabetes patients, excretion showed positive associations with HbA1c, serum glucose and eGFR, and inverse associations with diuretics, plasma betaine, dimethylglycine and glycine. In patients without diabetes mellitus and glucose <5.5 mmol/L, these associations of urinary excretion were weaker (HbA1c, glucose, diuretics, dimethylglycine, glycine) or inversed (eGFR, betaine). In the latter group, betaine excretion showed associations with age (positive), smoking (inverse) and plasma choline (positive) that were essentially absent in patients with diabetes. UACR was only weakly associated with betaine excretion, and the magnitude of correlation was independent of glucose metabolism.

**Table 3:** Spearman correlations of urinary betaine excretion at baseline.

	No diabetes						Diabetes	
	Glucose			Glucose				
	<5.5 mmol/L			≥5.5 mmol/L				
	(n = 978)			(n = 1154)			(n = 264)	
Sex (male)	−0.05			0.01			0.13	*
Age	0.14	***		0.16	***		−0.06	
Smoking (yes/no)	−0.16	***		−0.16	***		−0.08	
Body mass index	0.04			0.04			0.12	
Diuretics (yes/no)	−0.03			−0.04			−0.23	***
Plasma total homocysteine	−0.11	**		−0.12	***		−0.14	*
eGFR	−0.12	***		0.02			0.17	**
Serum glucose	−0.02			0.11	***		0.36	***
HbA_1c_	−0.02			0.03			0.39	***
Plasma betaine	0.14	***		0.03			−0.14	*
Plasma choline	0.18	***		0.11	***		0.11	
Plasma dimethylglycine	0.06			−0.06			−0.24	***
Plasma glycine	−0.02			−0.10	**		−0.20	**
UACR	0.09	**		0.11	***		0.08	

Abbreviatons: eGFR estimated glomerular filtration rate; UACR urinary albumin/creatinine ratio.

* p<0.05. ** p<0.01. *** p<0.001.

### Dose-response relationships for betaine excretion versus HbA1c, eGFR and plasma betaine

**Figure 2:**
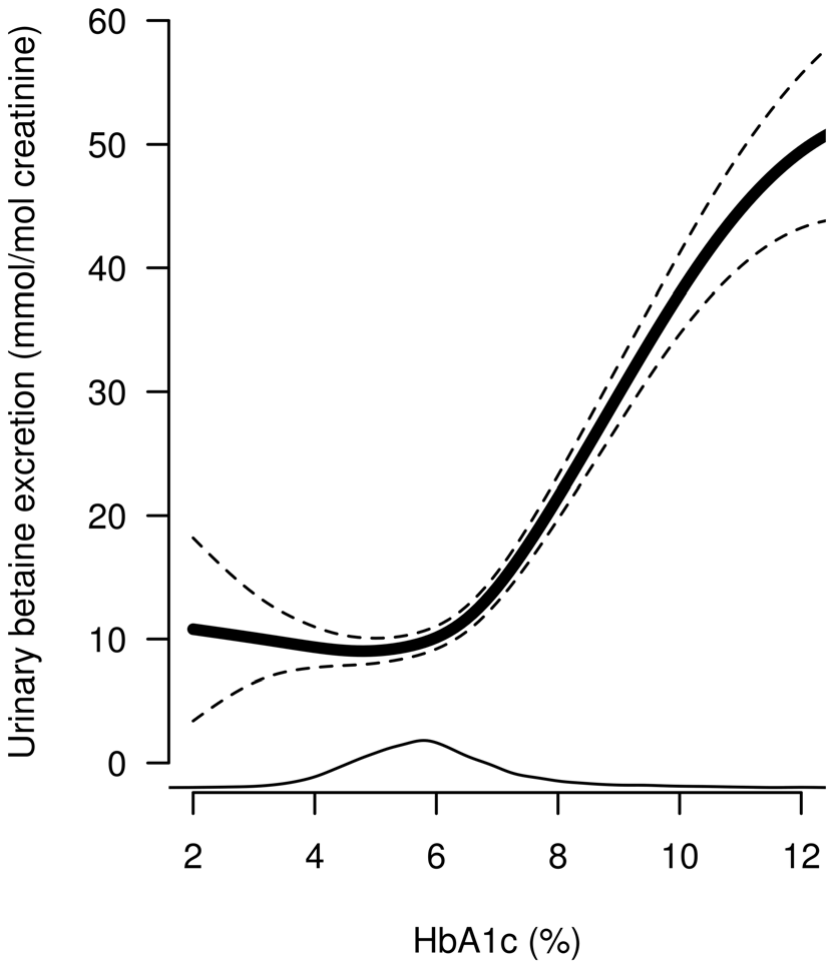
Generalized additive model showing the non-linear association between urinary betaine excretion and glycated hemoglobin (HbA1c). The dotted lines mark the 95% confidence interval. The line at the bottom indicates the distribution.

We used generalized additive models, adjusted for age and sex, to obtain graphical presentations of betaine excretion versus HbA1c, eGFR and plasma betaine. Urinary betaine showed a non-linear relationship with HbA1c; there was no relation below levels of about 6% and a strong positive relation at higher levels (Fig. 2). Using segmented regression, we identified a break-point (95% CI) of 6.5 (6.3–6.7)%, p<0.001. The regression coefficients were 0.5 (0.0–1.1) and 6.3 (5.8–6.9)%/mmol/mol creatinine below and above this break-point, respectively.

**Figure 3:**
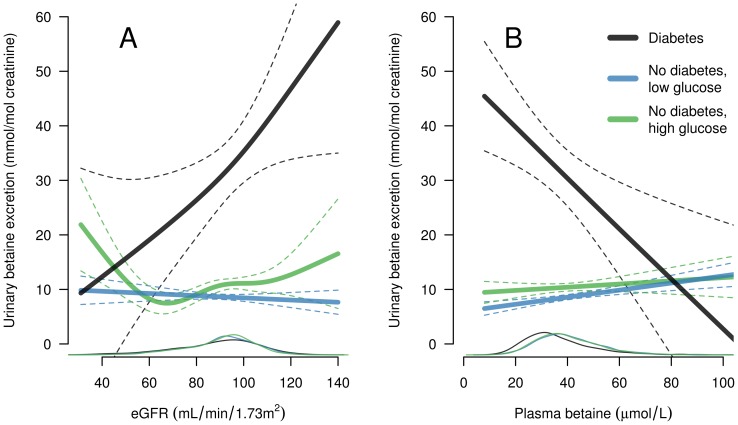
Generalized linear models showing age and gender adjusted associations between urinary betaine excretion and estimated glomerular filtration rate (A) and plasma betaine (B). Results, with 95% confidence intervals, for patients with diabetes mellitus (black line), no diabetes and serum glucose ≥5.5 mmol/L (green line), and no diabetes and serum glucose <5.5 mmol/L (blue line) are shown. Lines at the bottom indicate distribution.

We further investigated the dose-response relationship between betaine excretion and indices with contrasting associations in patients with and without diabetes, i.e. eGFR and plasma betaine. These associations were investigated separately in patients with diabetes, patients without diabetes and glucose ≥5.5 mmol/L and patients without diabetes and glucose <5.5 mmol/L (Fig. 3). eGFR showed a positive relation to betaine excretion in diabetes patients, and the curve was linear throughout the entire eGFR distribution. In patients without diabetes but with glucose ≥5.5 mmol/L there was a curvilinear relationship with an upward slope with broad confidence intervals at the extreme eGFR values. In patients without diabetes and with glucose <5.5 mmol/L, a weak inverse relationship was observed (Figure 3 A). Plasma betaine showed an inverse relation to betaine excretion in diabetes mellitus patients, and the curve was linear through the whole plasma betaine distribution, whereas the curves for both non-diabetic groups showed a slightly positive relationship (Figure 3 B).

### Determinants of urinary betaine excretion in patients with diabetes

We investigated associations between betaine excretion and baseline characteristics in diabetes mellitus patients by linear regression analyses (Table 4). Age (ß = 0.13) and gender (ß = 0.20) were moderate predictors. In a simple model adjusting for age and gender, the strongest associations were observed for HbA1c (positive), antidiabetic combination therapy (positive), eGFR (positive), diuretics (inverse) and plasma betaine (inverse), in that order. Several of these associations remained after multivariable adjustments and the strongest determinant was HbA1c (ß = 0.34). We found no significant difference in betaine excretion according to type of antidiabetic medication (data not shown). However, diabetes mellitus patients (n = 120) treated with any combination of antidiabetic drugs (metformin, sulfonylurea, insulin, acarbose, thiazolidinediones) had higher median (IQR) excretion (42.1 (58.8) mmol/mol creatinine) than patients (n = 62) on mono-therapy (21.6 (31.0) mmol/mol creatinine) (p = 0.004) or those (n = 60) taking no drugs (14.6 (22.5) mmol/mol creatinine) (p<0.001).

**Table 4:** Determinants of urinary betaine excretion in diabetes by linear regression.

	Adjusted for gender and age			Fully adjusted^a^			
	β^b^	P value		β^b^		P value	
Gender (male)	0.12	0.058		0.20		0.001	
Age	−0.06	0.31		0.13		0.094	
Diuretics (yes/no)	−0.22	<0.001		−0.18		0.003	
Mono therapy c	0.14	0.052		0.04		0.53	
Combination therapy c	0.35	<0.001		0.26		<0.001	
eGFR	0.24	0.004		0.10		0.24	
HbA1c	0.36	<0.001		0.34		<0.001	
Plasma betaine	−0.15	0.017		−0.09		0.13	
Explained variance (R^2^)					0.27		

Abbreviations: eGFR estimated glomerular filtration rate.

a Adjusted for all variables in the model.

b Standardized beta-coefficient.

c Antidiabetic medication vs no antidiabetic medication.

Not significant in age and gender adjusted analysis: body mass index, smoking, hypertension, type of diabetes, total cholesterol, high density lipoprotein cholesterol, low density lipoprotein cholesterol, apo lipoprotein A1, triglycerides, urinary albumin/creatinine ratio.

### Initial assessment of urinary betaine as a diagnostic tool

We evaluated the discriminatory ability of urine betaine to identify patients with diabetes mellitus, using ROC methodology. Subjects without diabetes and with glucose <5.5 mmol/L were used as reference population. The AUC for betaine excretion at baseline was 0.82 (0.79−0.86), suggesting good discriminatory ability.

We also determined the test-retest stability of betaine excretion. This was obtained by longitudinal measurements of excretion in all patients (n = 2120) with urine samples donated at least once during trial follow-up over median (IQR) 39 (17) months, and calculated as the coefficient of reliability (CoR). The CoR of betaine excretion for the entire cohort was 0.73 and for patients randomized to placebo treatment 0.74. In comparison, the CoR of UACR was 0.67. In diabetes patients (n = 221), the CoR of betaine excretion was 0.70.

We finally studied urinary betaine excretion as a predictor of future diabetes development, with a median follow-up time of 39 months (Table 5). Using logistic regression with self-reported new diabetes as the outcome, each standard deviation increase in log-transformed urinary betaine levels was associated with a hazard ratio (HR) (95% CI) of 1.60 ((1.21, 2.12), p = 0.001) in an age and sex adjusted model, and a HR of 1.65 ((1.24, 2.20), p = 0.001) in a multivariate model. When patients with pathologically elevated glucose values during follow-up were included in the endpoint, urinary betaine was a somewhat weaker predictor, but still significant. Apart from betaine excretion, only body mass index and uric acid were significant determinants in the multivariate models, whereas HbA1c was not (data not shown). Further, exclusion of HbA1c only marginally strengthened urinary betaine as predictor. In fasting patients (n = 723, events = 18) a similar result was observed in the multivariate model with the combined endpoint of self reported diabetes or pathologically elevated glucose values (HR 1.71 (1.08, 2.71)), p = 0.023). If all patients with HbA1c≥6.5% were excluded (n = 428), solely body mass index remained a significant determinant (data not shown). Fractional betaine clearance and plasma betaine were not associated with risk of developing diabetes (data not shown).

**Table 5:** Baseline urinary betaine excretion as a determinant of new diabetes per standard deviation increase^a^.

	Age and sex adjusted				Multivariate adjusted^b^			
	N	Events	HR (95% CI)	P value	N	Events	HR (95% CI)	P value
Endpoint 1^c^	2076	38	1.60 (1.21, 2.12)	0.001	2038	38	1.65 (1.24, 2.20)	0.001
Endpoint 2^d^	2076	75	1.34 (1.08, 1.67)	0.008	2038	74	1.37 (1.10, 1.71)	0.005

a Patients with no diagnosis of diabetes and either fasting glucose <7.0 mmol/L or non-fasting glucose <11.1 mmol/L at baseline.

b Adjusted for age, gender, statin treatment, glycated haemoglobin, triglycerides, uric acid, urinary albumin creatinine ratio, body mass index.

c Self-reported diabetes during follow-up.

d Self-reported diabetes and/or fasting glucose ≥7.0 mmol/L or non-fasting glucose ≥11.1 mmol/L during follow-up.

## Discussion

### Principal findings

In this study of 2396 patients undergoing coronary angiography for mainly stable angina, the strongest determinants of urinary betaine excretion were diabetes mellitus, age and eGFR. We observed a strong non-linear association to HbA1c, with an inflection point at 6.5%, which has been introduced as cut-off for the diagnosis of diabetes mellitus [23]. Excretion showed divergent associations with key metabolic and clinical indices when comparing patients with diabetes to subjects without diabetes, which is in line with diabetes being a strong effect modifier. The discriminatory power of betaine excretion for diabetes corresponded to an AUC by ROC analysis of 0.82, and the test-retest stability of betaine excretion over median 39 months was high. Notably, betaine excretion was a strong, independent predictor of the development of new diabetes. These results point to a potential of betaine excretion in risk assessment and follow-up of diabetes.

### Determinants of urinary betaine excretion

The results from the present study extend and mostly confirm the observations on betaine excretion previously made in smaller populations by Lever et al [1], [3], [4], [7], [24], [25]. They reported on a massive increase and greater variability in excretion in patients with diabetes compared to healthy subjects, of similar magnitude as reported here [3], [4], [24], [25]. They also found a positive correlation to HbA1c [4] and an increase with age [7]. Recently, in two small cohorts of patients with diabetes mellitus, urinary betaine was shown to correlate strongly with urinary glucose excretion [25], [26]. Lever et al also observed an association between betaine excretion and cardiovascular disease in patients with lipid disorders [1], and increased risk of heart failure both in patients with high and low betaine excretion [27].

The associations of betaine excretion with eGFR, plasma betaine and diuretic medication in the present study have not previously been reported, possibly because of the pronounced effect modification of the diabetic state itself. The absence of an association with HbA1c below 6−6.5%, a level which divides patients with risk low risk of progression to diabetes from those with increased risk of diabetes complications [23], indicates that betaine excretion may be used for diagnostic purposes or as a risk marker. Increased excretion in patients with antidiabetic medication, but with no difference between the various drugs, may reflect that medication serves as a proxy for disease severity.

### Metabolic ramifications and possible mechanisms

In subjects without diabetes, betaine excretion is essentially independent of eGFR >50 mL/min/1.73 m^2^ and of plasma betaine, which may reflect that the fractional clearance is low (less than 2.0%) in healthy subjects [9]. In contrast, the positive relation between betaine excretion and eGFR across its entire distribution in diabetes patients might suggest impaired tubular handling of betaine. This is in agreement with the previous observation of betaine excretion being associated with excretion of retinol binding protein [4], an early marker of tubular injury [28]. Tubular changes are apparent before the onset of glomerular permeability dysfunction in diabetes [29], which is consistent with the weak association between betaine excretion and albuminuria in our study. The fact that tubular glucose uptake is independent of insulin may render the tubuli particularly susceptible to potentially damaging effects of high glucose [30], and the strong correlation between betaine excretion and HbA1c raises the possibility that long-term glucose toxicity may affect tubular betaine handling and thereby cause high betaine excretion in diabetes.

Another possible mechanism of high urinary betaine excretion is effusion of betaine from the renal tubuli or medulla. Betaine is an important osmolyte in the medulla, reaching concentrations of several magnitudes higher than in plasma. It is produced from choline in the cortex, mainly in the proximal tubule, and is accumulated in the medulla by the basolaterally, osmoregulated betaine/gamma aminobutyric acid transporter (BGT1) [31]. Renal secretion of betaine as a source of high excretion is supported by the observation by Lever et al of a fractional betaine clearance that exceeded 100% in patients with serious renal disease [3], [4]. Such patients were excluded in the current study, which may explain the lower values in our cohort. In patients with diabetes, betaine excretion has also been shown to correlate strongly with urinary sorbitol excretion. Sorbitol is an important renal osmolyte that is not involved in one-carbon metabolism, and high renal excretion of sorbitol could indicate effusion. We did not measure sorbitol, but the strong correlations between urinary betaine and urinary dimethylglycine and sarcosine, which are not important osmolytes [31], argues against renal secretion as a dominant determinant [3]. Other causes of increased betaine excretion include fibrate therapy [24], but no patients in the current investigation were taking such medication. Also, some foods containing proline betaine may increase excretion [32]. Our observational data do not allow any conclusion on the underlying pathomechanisms affecting renal handling of betaine in patients with diabetes.

Chronic, but not acute, increased diuresis has been shown to impair betaine reuptake [11]. We found that diuretic medication reduced betaine excretion in patients with diabetes mellitus. We also observed an almost 20% reduction in eGFR in diabetic patients treated with diuretics (data not shown), but the relation between betaine excretion and diuretic medication was upheld after adjustment for eGFR in a multivariate model. Conceivably, commonly used diuretics may alter betaine reabsorption in diabetes through their effects on ion transporters and water reuptake in the renal tubules [33].

### Urinary betaine as a diagnostic tool

The marked, more than three-fold, difference in median urinary betaine excretion between subjects with and without diabetes mellitus motivated the initial assessment of betaine excretion as an indicator of diabetes. In ROC analyses, an AUC value of 0.82 was obtained, which indicates that betaine excretion has good discriminatory power.

We estimated a CoR of 0.73 by 2−3 measurements over median (IQR) 39 (17) months. The high long-term coefficient of reliability of betaine excretion observed in the present study suggests that an individual's excretion is relatively constant over several years, and that a single measurement will correctly classify the study participant with respect to the average excretion over time. The observation that urinary betaine excretion is highly individual is in accordance with results published by Lever et al. [5], [6], [34], who have also shown that excretion is not affected by osmotic stress [6], and that collecting 24-hour urine samples does not seem to add to the information provided by the betaine/creatinine ratio [6], [7].

Urinary betaine excretion was an independent predictor of the development of new diabetes, with a log-linear risk relationship, and remained significant when only fasting patients were included in the analyses. The estimates were largely independent of adjustment for HbA1c. However, excluding all patients with HbA1c ≥ 6.5% attenuated the association, which reflects the strong relation between urinary betaine and HbA1c above this cut-off. Incidentally, the same cut-off has recently been included as a diagnostic test for diabetes based on the increased risk of retinopathy above this level, but there is limited data showing the potency of such a cut-off to predict macrovascular complications [35]. Taken together, this suggests that HbA1c is not a panacea in patient evaluation at the early stages of disturbed glucose metabolism, and leaves room for other diagnostic and prognostic modalities. Although the estimates in this study are based on a modest number of endpoints, our findings indicate that increased betaine excretion is a sensitive marker of incident diabetes mellitus.

### Strengths and limitations of the study

Our data include a large number of study participants delivering both urine and plasma samples on two or three occasions over several years, and betaine and related methylamines were measured in both matrices. The study size allows precise effect estimates in subgroups and to assess the long-term test-retest stability of betaine excretion. To measure renal function, we used GFR estimated according to the CKD-EPI procedure; it is superior to measurement of serum creatinine but less accurate than clearance values of exogenous filtration markers [36]. A limitation of the study is the fact that the majority of patients were not fasting, which weakens the diagnostic accuracy of serum glucose to identify diabetes. We chose to analyse type 1 and type 2 diabetes combined, as they behaved similarly in all analyses conducted. However, given the small number of patients with type 1 diabetes and the different pathogeneses, we must be cautious when generalizing our results. The low number of patients who developed diabetes according to our criteria, and the lack of standardized testing for diabetes, also prevent us from drawing firm conclusions on the predictive potential of urinary betaine. We did not have measurements of HbA1c beyond baseline. Another limitation is the lack of data on urine glucose, which might have enlightened mechanistic aspects. Finally, because the majority of our patients were treated with statins, which may influence diabetes development [37], our results need to be confirmed in population based studies.

## Conclusions

Diabetes mellitus and long-term glycemic control are the strongest determinants of urinary betaine excretion in a population of patients with mainly stable angina pectoris and without severe renal failure. Because the long-term test-retest stability is high and betaine excretion also is associated with an increased risk of new diabetes development, our data should motivate studies on the utility of betaine excretion in risk assessment and follow-up of diabetes in the general population.
